# A Plea for the Eye

**Published:** 1906-07-28

**Authors:** 


### A Plea for the Eye.

Among the matters considered by the General Medical Council during its session in May last were two communications from the Privy Council, referring respectively to a petition of the "British Optical Association" for the grant of a Charter of Incorporation, and to a Bill entitled "The Sight Testing Opticians Bill," which is now before the House of Lords. The petition and the Bill were both submitted to the Medical Council "for any observations which they might have to make thereon." The Council dealt with both in a single memorandum—originally submitted by the President and adopted with some slight modifications—the general drift of which was to show that, until the second half of the nineteenth century was well advanced, the selection of spectacles, which had until then been made by the persons needing them, or had remained in the hands of traders styling themselves "opticians," had been entirely empirical and devoid of any guidance from science. The very nature of the conditions or changes which caused spectacles to be required were entirely mistaken or misunderstood, and it was not until the questions at issue were investigated by medical practitioners, whose knowledge of optics was reinforced by a knowledge of physiology, that the truths now known concerning refraction and ocular defects were ascertained. The memorandum further pointed out that functional and refractive errors of the eye were frequently productive of, or associated with, conditions of actual or of impending disease, and that the bestowal of "sight-testing" diplomas upon persons not medically educated, and who would be likely to use them as instruments of trade advertisement, could hardly fail to lead to disastrous results. We do not imagine that the petition is likely to be granted, or that the Bill is likely to receive the sanction of Parliament. Spectacle sellers are now describing themselves by a variety of absurd nicknames, as "optologists" and the like, and are advertising in all directions their competence to deal with all sorts of affections of the sight. It is high time that the Medical Council should strike at the root of their unfounded pretensions by taking measures to secure that an adequate knowledge of ophthalmology should be possessed by every qualified medical practitioner, and that this important branch of the science and art of healing should no longer be regarded, either by the public or by the profession, as one falling almost exclusively within the domain of specialism.

The dangers likely to arise from the present state of public opinion are mainly that serious disease is likely to be overlooked while time is being wasted in the correction of ocular defects of no real importance. The human eye, as Helmholtz pointed out, is a very defective instrument; insomuch that, if it were sent home to us by an optician, we should be likely to return it upon his hands. Its proportions are often faulty, its media are often spotty, its curvatures are seldom correct, and frequently are not concentric. In spite of all this, it may be an extremely useful organ, and may be quite sufficient to fulfil all the requirements of its owner. But if the owner should be suffering from symptoms which it is at all possible to ascribe to "eye strain," and if he should then seek advice from a spectacle seller ignorant of medicine, such a person would usually have no difficulty in finding out "ocular defects" sufficient, in his want of judgment, to explain the symptoms, and would often hold out valiant expectations of cure by the use of glasses. Some time would be required in order to demonstrate their uselessness, and disease might meanwhile make steady progress. The Medical Council acutely suggested that, instead of its being left to spectacle sellers to discover "disease," and to refer their would-be customer to a surgeon, the spectacle sellers should be prohibited from "prescribing" glasses until the subject had obtained a certificate of freedom from disease from an ophthalmologist. When any sufferer goes to the spectacle seller the latter tries to find some ocular defect on which the symptoms may possibly depend. When the sufferer goes to a medical practitioner he endeavours to find the correct explanation of the symptoms, either in the eyes or elsewhere as the case may be. The spectacle seller approaches the case from a narrow and limited view, the medical man from a wide and general standpoint. In many cases the spectacle seller practises upon the ignorance of his customer, and supplies him with a superfluous assortment of different glasses at enormous prices, to be used for different purposes. The remedy is the steady assertion of its rightful position by the medical profession, coupled with such watchfulness over education as to secure that the assertion may be at once justified and successful.

